# STAT3 controls IL6-dependent regulation of serotonin transporter function and depression-like behavior

**DOI:** 10.1038/srep09009

**Published:** 2015-03-11

**Authors:** Eryan Kong, Sonja Sucic, Francisco J. Monje, Sonali N. Reisinger, Giorgia Savalli, Weifei Diao, Deeba Khan, Marianne Ronovsky, Maureen Cabatic, Florian Koban, Michael Freissmuth, Daniela D. Pollak

**Affiliations:** 1 Department of Neurophysiology and Neuropharmacology, Center for Physiology and Pharmacology, Medical University of Vienna; 2 Department of Pharmacology, Center for Physiology and Pharmacology, Medical University of Vienna

## Abstract

Experimental evidence suggests a role for the immune system in the pathophysiology of depression. A specific involvement of the proinflammatory cytokine interleukin 6 (IL6) in both, patients suffering from the disease and pertinent animal models, has been proposed. However, it is not clear how IL6 impinges on neurotransmission and thus contributes to depression. Here we tested the hypothesis that IL6-induced modulation of serotonergic neurotransmission through the STAT3 signaling pathway contributes to the role of IL6 in depression. Addition of IL6 to JAR cells, endogenously expressing SERT, reduced SERT activity and downregulated SERT mRNA and protein levels. Similarly, SERT expression was reduced upon IL6 treatment in the mouse hippocampus. Conversely, hippocampal tissue of IL6-KO mice contained elevated levels of SERT and IL6-KO mice displayed a reduction in depression-like behavior and blunted response to acute antidepressant treatment. STAT3 IL6-dependently associated with the SERT promoter and inhibition of STAT3 blocked the effect of IL6 *in-vitro* and modulated depression-like behavior *in-vivo*. These observations demonstrate that IL6 directly controls SERT levels and consequently serotonin reuptake and identify STAT3-dependent regulation of SERT as conceivable neurobiological substrate for the involvement of IL6 in depression.

An involvement of the proinflammatory cytokine interleukin 6 (IL6) in the pathophysiology of depression is suggested by converging evidence obtained from studies in human patients^1^,^2^ and respective animal models of the disease^3^,^4^. However, how IL6 impinges on neurotransmission herby modulating the behavioral output of the brain, remains largely unknown. The serotonin transporter (SERT, SLC6A4) is the principle site of action of the most commonly prescribed antidepressant drugs (selective serotonin reuptake inhibitors, SSRIs) and SERT activity shapes serotonergic neurotransmission, which is implicated in the behavioral features and pathophysiology of depression^5^,^6^.

To further explore existing evidence that some proinflammatory cytokines (e.g., TNFα and IL1β)^7^,^8^,^9^,^10^ can modulate SERT activity, we tested the hypothesis that IL6-dependent activation of the STAT3 canonical inflammatory signaling^11^ exerts direct regulatory control over SERT expression, function and depression-like behavior.

To determine the effect of IL6 on SERT expression and function, human choriocarcinoma (JAR) cells, endogenously expressing SERT, were treated with IL6 for 48 hours. A significant reduction in the specific uptake of [^3^H]5-HT and a marked reduction in maximal transport velocity (V_max_), without a decrease in 5-HT affinity were observed (Figure 1a and 1b). A commensurate decline in SERT mRNA and protein levels was found (Figure 1c and 1d).

We next recapitulated the dampening effect of IL6 on SERT expression in the neuronal system *in-vitro*, by incubating mouse primary neurons with IL6 (Figure 1e) and *in-vivo*, by central (intracerebroventricularly; i.c.v.) application of IL6 (Figure 1f), a treatment previously shown to induce depression-like behavior in mice^4^. A corresponding increase in SERT mRNA and protein levels was observed in the brains of mice deficient in IL6 (IL6-KO mice) (Figure 2a and 2b) which was paralleled by enhanced [^3^H] citalopram binding to synaptosomal membranes (Figure 2c). No alterations in the levels of the closely related dopamine transporter (DAT) in IL6-KO mice were observed (Figure 2d). Altered SERT expression was associated with a significant reduction in depression-like behavior of IL6-KO in the Forced Swim Test (FST), the Sucrose Preference Test (SPT), the Novelty Suppressed Feeding Test (NSF) and blunted sensitivity to acute antidepressant treatment with the SSRI Escitalopram in the FST (Figure 2e–2g).

The herein reported reduction in despair-related immobility in the FST in IL6-KO is in agreement with previous reports^12^. The observed significant increase in sucrose preference in IL6-KO mice, which is indicative of less susceptibility to depression-related anhedonia, confirms an earlier description of enhanced sucrose consumption of IL6-KO mice^12^. This potential resilience of IL6-KO mice is also in line with the described resistance of IL6-KO mice to the induction of a depression-like phenotype, verified in two independent animal models^3^,^12^. While a direct causal relationship between elevated levels of SERT and the altered depression-related phenotype in IL6-KO mice cannot be established in the present study, our observations of augmented SERT expression in IL6-KO mice strikingly mirror image the reported depression-like behavior characteristics of SERT-deficient mice (SERT-KO)^13^. These results suggest that - contrary to what is expected given the dampening effects of SSRIs on SERT activity and their role as pharmacological antidepressants - depression-like behavior could be associated with decreased SERT levels. This hypothesis is further supported by findings of reduced SERT expression in two independent stress-based animal models of depression^14^,^15^.

To unveil the regulatory principle mediating the effects of IL6 on SERT levels and depression-like behavior, the relevance of the STAT3 signaling cascade - the predominant mechanism by which transcriptional control upon IL6-receptor activation is exerted^11^ -was investigated *in-vitro* and *in-vivo*. Incubation of JAR cells with IL6 resulted in increased levels of active, phosphorylated STAT3 (Figure 3a and 3b) and blockage of the IL6 -receptor with the monocolonal antibody tocilizumab and of STAT3 by stattic (a small-molecule inhibitor of STAT3 activation and dimerization^16^), blunted the effect of IL6 on [^3^H]5-HT uptake (Figure 3c). Assuming that the increase of phosphorylated STAT3 was directly relevant to the regulation of SERT expression, STAT3 ought to reside on the SERT promoter. This prediction was tested using chromatin immunoprecipitation (ChIP) which revealed binding of STAT3 to the SERT promoter under basal conditions together with a substantial enhancement in IL6 treated JAR cells (Figure 3d). Finally, we set-out to examine the direct involvement of STAT3 in depression-like behavior and found that – as expected – pharmacological inhibition of STAT3 elevated SERT expression and reduced depression-like behavior in wild-type mice (Figure 3e–g).

In the present study behavioral performance and gene expression were evaluated in different cohorts of animals, since prior testing, specifically using behavioral tests associated with acute stress exposure (such as the FST), could bias subsequent molecular analyses, as shown for several proteins, including SERT^17^. Hence this design does not allow investigating a potential correlation between immobility in the FST and hippocampal SERT expression. Interestingly however, a recent study investigating behavioral despair in the FST and hippocampal SERT expression in different mouse strains did not reveal a correlation between SERT expression and immobility in the FST, neither at baseline nor after Fluoxetine treatment^18^.

In summary, results of the present study firstly demonstrate the direct regulatory constraint of IL6-induced STAT3 signaling on SERT expression, function and depression-like behavior in the mouse (Figure 4).

While previous experiments have documented that other cytokines, such as IL-1β and tumor necrosis factor-α (TNF-α), can modulate SERT activity in the mouse brain^9^, these effects occur at the posttranslational level^8^,^9^,^19^. Our data collectively propose a novel concept in which the immune system, through activation of a canonical signaling pathway, exerts control over the expression of a neurotransmitter transporter herby participating in the modulation of the behavioral output of the brain.

## Methods

### Materials

[^3^H] 5-HT (28.1 Ci/mmol) and [^3^H] Citalopram (85.6 Ci/mmol) were purchased from Perkin Elmer (Boston, MA, USA). Cell culture media, supplements and antibiotics were all purchased from Invitrogen Corporation (Carlsbad, CA, USA). Human and mouse recombinant IL6 were obtained from eBioscience (San Diego, CA, USA), Stattic and Escitalopram were supplied by Sigma (Sigma Aldrich, Vienna, Austria), Tocilizumab was obtained from Roche (Vienna, Austria). Primary antibodies used were anti-STAT3 (Cell Signaling, #9139, Boston, MA, USA), anti-phospho-STAT3 (Cell Signaling, #9145), anti-beta-Tubulin (AbFrontier, #LF-MA20056, Seoul, Korea), and anti-SERT (Santa Cruz Biotechnology Inc., # Sc-1458, Santa Cruz, CA, USA). Secondary antibodies used were goat anti-rabbit IgG (Cell Signaling, #7074), rabbit anti-goat IgG (Santa Cruz Biotechnology Inc., #Sc-2020) and rabbit anti-mouse IgG (Cell Signaling, #7076).

### Cell culture

Cells were maintained at 37°C, 5% CO2 humidified atmosphere on standard plastic culture ware. HEK293 cells stably expressing YFP-tagged wild type human SERT (HEK-SERT) were grown in Dulbecco's Modified Eagle's Medium (DMEM), supplemented with 10% fetal calf serum, 1% penicillin/streptomycin and geneticin. JAR cells (American Type Culture Collection (ATCC, catalog Nr. HTB-144™, Manassas, VA, USA) were cultured in RPMI 1640 medium, supplemented with 10% fetal calf serum and 1% penicillin/streptomycin. Postnatal mouse (day 0–3) hippocampal neurons were dissociated and cultured according to Nunez^20^ in the presence of glial support cultures^21^. For uptake assays, cells were seeded onto 48-well culture plates coated with poly-D-lysine, and treated with 50 ng/ml IL-6, 5 μM stattic (5 μM in 0.1% DMSO), 500 nM tocilizumab and combinations thereof.

### Uptake assays

[^3^H]5-HT uptake was measured as described previously^22^. In brief, culture medium was aspirated and JAR cells were washed twice with Krebs-HEPES buffer (KHB) at 25°C. Cells were then incubated for 10 min at 25°C with KHB in the absence or presence of 10 μM paroxetine (to determine non-specific uptake, which amounted to >30% of total uptake). [^3^H]5-HT (0.1 to 30 μM) was added 6 min for JAR cells. Uptake was terminated by rapidly washing the cells with KHB at 4°C. Cells were subsequently lysed in 1% SDS and assayed for [^3^H] content.

### Radioligand binding assays

IL6 KO and WT mice were decapitated and cortical tissue was dissected on ice, homogenized and SERT containing membranes were prepared in a buffer containing 10 mm Tris·HCl (pH 7.5), 1 mm EDTA, 2 mm MgCl2. Radioligand binding assays were carried out essentially as previously described^23^ using 2 nm [^3^H] citalopram and 10 μM paroxetine (to determine non-specific binding).

### Chromatin immunoprecipitation assay (ChIP)

ChIP was performed as previously described^24^ in IL6 treated (50 ng/ml, 48 h) and untreated control JAR cells. After immunoprecipitation, the supernatant was used directly as template for qRT-PCR. Selective primer pairs flanking the potential binding site of STAT3 at the SERT promoter were used: forward 5′GATTCGCATGGTTCGGTCCT3′ and reverse 5′TTACACCTGCCCCAAACACC3′.

Manipulations carried in the absence of the primary antibody (MockIP) were used to define the assay blank. The relative levels of STAT3 binding to the SERT promoter were determined by qRT-PCR; the background (i.e., amplicons produced in the absence of a specific immunoprecipitation) was set 1. For the calculation of signal ratio, R, the following formula was used. R = exp_2_(CT^mock^ − CT^specific^), where CT^mock^ and CT^specific^ are mean threshold cycles of qRT-PCR carried out in triplicate on DNA samples from immunoprecipitations in the absence (mock) and presence of the STAT3-directed antibody, respectively.

### Real time polymerase chain reaction (qRT-PCR)

Cultured cells were washed briefly twice with ice cold PBS and brain tissue was powderized in liquid nitrogen and then processed for RNA extraction. RNA isolation, cDNA synthesis and qRT-PCR analysis were carried out as previously described^19^. Relative mRNA expression of target genes was calculated as ΔΔCt values against that of control samples. The levels of β-actin mRNA was used to calculate ΔCt values for all samples. The following primer sequences were used: β-actin forward 5′ATGGTGGGAATGGGTCAGAAG3′ and reverse 5′TCTCCATGTCGTCCCAGTTG3′; SERT forward 5′GCTGAGATGAGGAACGAAGAC3′ and reverse 5′AGGAAGAAGATGATGGCAAAG3′.

### Western Blotting

Cultured cells were washed twice with ice-cold PBS; brain tissue was pulverized in liquid nitrogen and homogenized in a protein lysis buffer containing of 10 mM Tris-HCl, pH 7.5, 150 mM NaCl, 1% SDS, 0.5% Triton ×100, 1 mM EDTA, 10 mM NaF, 5 mM Na_4_P_7_O_2_, 10 mM Na_3_VO_4_ and protease inhibitor cocktail (Complete™, Roche Diagnostics, Mannheim, Germany). Protein isolation, quantification and Western Blot analysis followed a previously described protocol^19^. Quantification was performed by chemiluminescent imaging with a FluorChem HD2 (Alpha Innotech, San Leandro, Calif., USA) using the respective software. Values obtained from densitometry of target proteins were normalized to those of the housekeeping protein β-tubulin for the same samples.

### Animals and Housing

Male C57Bl6/N were purchased from Charles River (Sulzfeld, Germany), male IL6 knock-out mice on a C57Bl/6J background (strain 002650) and wild-type control mice, 10–12 weeks old, were obtained from Jackson Laboratories (Bar Harbor, ME, USA). All animals were naïve, i.e without any prior manipulation, at the onset of experiments. Animals were housed in a temperature-controlled colony room (22 ± 1°C) and provided with food and water *ad libitum* unless stated otherwise. Mice were maintained on a 12 hours light/dark cycle (with lights on at 6:00 a.m., 200–220 lux inside the cages). Sample sizes used were similar to those reported in previous studies^4^,^25^,^26^ and with the aim to reduce animal suffering and keep the number of animals used at the minimum level. Animal experiments described in this study were approved by the national ethical committee on animal care and use (Bundesministerium für Wissenschaft und Forschung) and carried out according to EU-directive 2010/63/EU.

#### Behavioral tests

Animals were single-housed in standard transparent laboratory cages one week prior to the start of behavioral experiments, which were all carried out during the light-phase of the light/dark cycle. Behavioral analyses were carried out by an experimenter blinded to the experimental groups.

#### Drug treatment

Escitalopram was dissolved in 0.9% NaCl and administered by intraperitoneal (i.p.) injection at a dose of 10 mg/kg in 0.25 mL. Control animals received 0.9% NaCl injections (i.p.). Behavioral testing was carried out 30 min after drug treatment. Stattic was dissolved in DMSO and diluted in 0.9% NaCl and administered by (i.p.) injection at a dose of 5 mg/kg in 0.25 mL. Control animals received equal amount of DMSO diluted in 0.9% NaCl (i.p.). Behavioral testing was carried out 24 hrs after drug treatment.

#### Forced swim test

The forced swim test (FST) was carried out as previously described during the light phase of the day^9^. Briefly, mouse behavior was tracked using an infrared video camera and monitored by VIDEOTRACK [PORSOLT] software (Viewpoint®, France). The test chamber consisted of a Plexiglas beaker (diameter: 19 cm, depth: 23 cm), filled with tap water (23–25°C). The test had a total duration of 6 min of which the last 4 min were used for the analysis of immobility. Percentage of immobility was calculated as the amount of time (in sec) the animal spent immobile during the total evaluation period (240 sec). Immobility is defined as cessation of all movements except the minimum postural adjustments required for maintaining the nostrils above the surface of the water to allow for breathing^26^.

#### Sucrose preference test

The SPT test was carried out essentially as described by Khan et al.^27^. Briefly, during a 4 days training phase, mice were habituated to drink a 2% sucrose solution. The day before the sucrose preference test mice were deprived of food and water for 18 hours. During the test, subjects were given a free choice between two bottles, one with the sucrose solution and the other with water. Mice were tested over 3 h, starting at 9:00 a.m. To prevent possible effects of side preference in drinking behavior, the position of the bottles (right/left) was alternated between animals. Total liquid consumption was measured by weighing the bottles before and after the SPT. Sucrose preference was calculated as percentage of sucrose solution consumed relative to the total amount of liquid intake.

#### Novelty-Suppressed Feeding Test

The novelty-suppressed feeding (NSF) paradigm was performed according to a previous study^28^ with minor modifications. The testing apparatus consisted of a clear Plexiglas arena (33 × 47 × 17 cm), brightly lit (800 lux). Because our mice were single housed, the control test of 5 min food consumption was carried out in each mouse home cage, placed aside to the NSF arena, in dim light (30 lux).

#### Brain dissection

Mice were sacrificed by neck dislocation and brains were rapidly dissected on ice. Isolated tissues were stored in RNAlater® (Ambion, Austin, TX, USA) for RNA isolation experiments or immediately immersed in liquid nitrogen and kept at −80°C for protein isolation.

#### Central administration of IL-6

Recombinant mouse IL6 was purchased from Invitrogen (MD, USA) and diluted in artificial CSF (124 mM NaCl, 2.5 mM KCl, 2.0 mM MgSO4, 1.25 mM KH2PO4, 26 mM NaHCO3, 10 mM glucose, 4 mM sucrose, 2.5 mM CaCl2) containing 0.1% bovine serum albumin (BSA) as carrier protein. Ten weeks old male C57Bl/6N mice were used for this experiment. At the time of surgery, mice were anesthetized using a mix of ketamine (Ketanest®, Pfizer Corporation Vienna, Austria, 100 mg/kg) and xylazine (Rompun®, Bayer Vienna, Austria 20 mg/kg) mix administered i.p. and fitted with a stainless-steel guide cannula (26 gauge; Plastics One, Bilaney, Germany) aimed at the lateral ventricle. Coordinates relative to skull at bregma: anterior–posterior, −0.26 mm; mediolateral, −1.0 mm; dorso-ventral, −2 mm (relative to surface of the skull). Post-surgery analgetic treatments and applied and mice were transferred to individual cages and handled daily for 3 min per day during a seven days recovery period. 3 μl of IL-6 (1 μg total) or CSF solution was manually injected into the lateral ventricle over a 2 min period (rate of infusion at 1.5 μl min-1). The infusion cannula remained in place for an additional 5 min to prevent backflow leakage. Mice were sacrificed 24 hours after injection and hippocampi were collected for Western Blot analysis. The position of the intracerebroventricular (i.c.v.) cannula was verified in each case by coronal sectioning and histological analysis (Nissl staining) at the completion of each experiment.

### Data collection and statistical analysis

**Figure 1:**
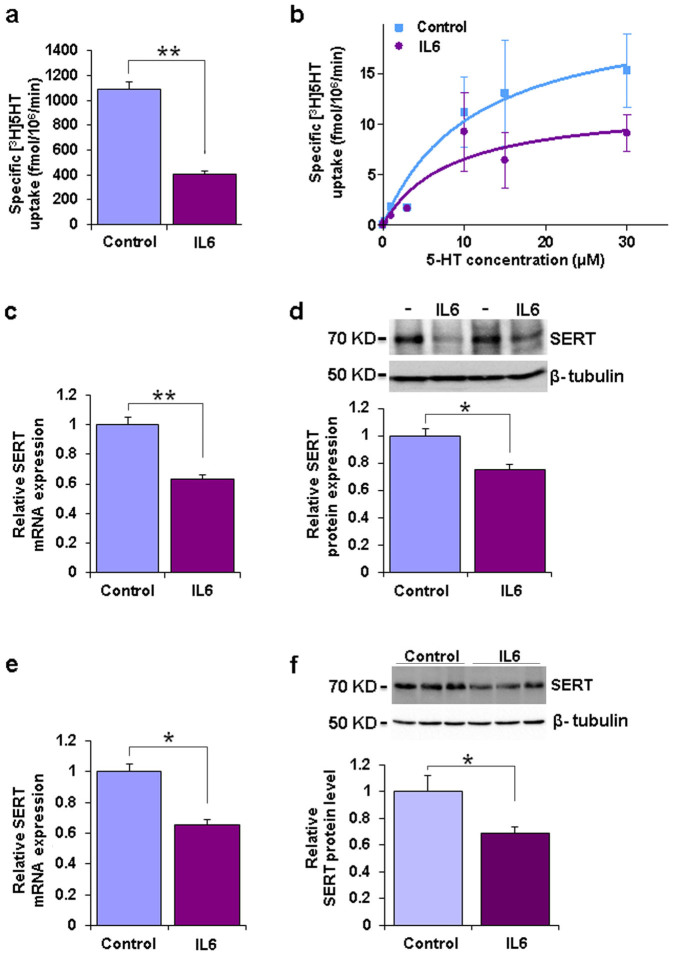
SERT expression is modulated by IL6 *in-vitro* and *in-vivo*. (a) JAR cells (5 * 10^5^ cells) were incubated for 48 h either in the absence (control) or presence of IL6 (50 ng/ml). The activity of SERT was quantified by measuring specific cellular uptake of 0.1 μM [^3^H]5-HT (p = 0.0001; t*_(11)_* = 6.308; n = 9 per group). (b) Kinetic characterization of [^3^H]5-HT uptake in JAR cells: K_m_ values were 7.39 ± 2.24 μM (control) and 3.70 ± 1.46 μM (IL6); the V_max_ values were 23.2 ± 7.9 (control) and 11.9 ± 4.1 pmol/10^6^ cells/min (IL6). (c) SERT mRNA (qRT-PCR) (p = 0.0024; t*_(10)_* = 5.676; n = 5–6 per group) and (d) protein levels (Western Blot) (p = 0.0362; t*_(7)_* = 3.622; n = 4 per group) in untreated control and IL6 treated JAR cells. The blot is a representative of four independent experiments and blot images were cropped for comparison. (e) SERT mRNA levels in untreated (control) and IL6 treated (50 ng/ml, 48 h) primary mouse hippocampal neurons (p = 0.0347; t*_(11)_* = 2.541; n = 6 per group). (f) SERT protein expression in hippocampal tissue of control and IL6 injected (i.c.v.) mice (p = 0.0418; t*_(9)_* *= * 5.272; n = 4 to 6 per group). Data are depicted as mean +/− SEM. * p < 0.05, ** p < 0.01.

**Figure 2:**
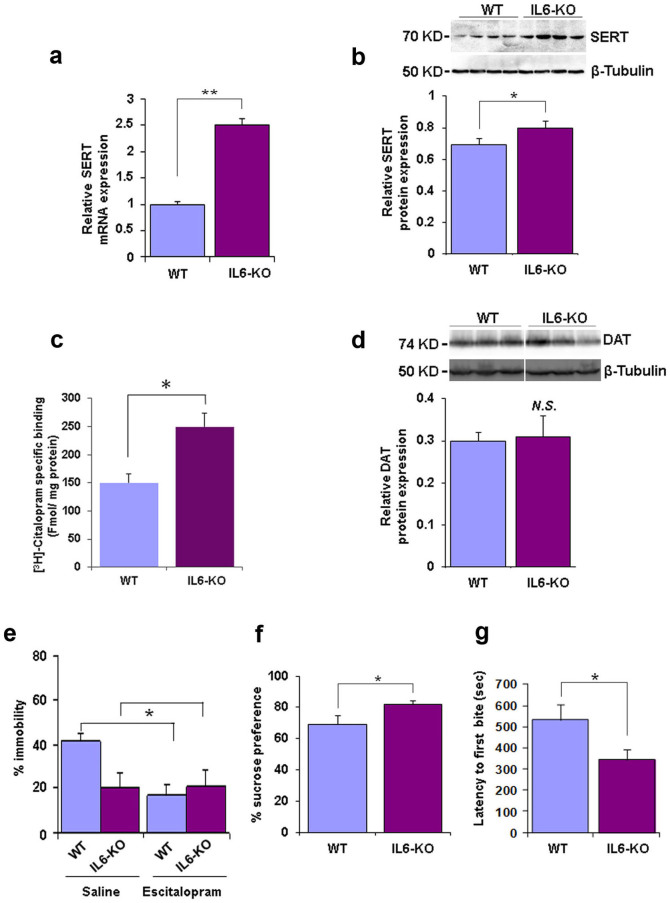
SERT expression and depression-like behavior in IL6-KO mice. (a) SERT raphe nuclei mRNA levels (qRT-PCR) (p = 0.0032; t*_(7)_* = 3.984; n = 4 per group), (b) SERT hippocampal protein (p = 0.0231; t*_(7)_* = 3.236; n = 4 per group), (c) radioligand binding assays with the selective SERT ligand [^3^H]citalopram (2 nm) on synaptosomal membranes prepared from cortical tissue (p = 0.010; t*_(17)_* = 2.885; n = 8–10 per group) and (d) DAT striatal protein levels (p = 0.1428; t*_(7)_* = 1.737; n = 4 group) in wild type (WT) and IL6-KO mice. The blots are each representative of four independent experiments and blot images were cropped for comparison (e) Percentage of time spent immobile and response to acute injection of Escitalopram (and saline control) in the Forced Swim Test (main effect of strain *F*_(2,17)_ = 4.99, p = 0.0423, main effect of treatment *F*_(2,17)_ = 4.52, p = 0.0523, strain x treatment interaction *F*_(2,17)_ = 9.41, p = 0.0083; n = 4 to 5 per group), (f) relative sucrose preference in the Sucrose Preference Test (p = 0.0461, t*_(17)_* = 2.151, n = 9 to 10 per group) and (g) latency to feed in the Novelty Suppressed Feeding test (p = 0.0134, t*_(17)_* = 2.76, n = 9 to 10 per group). Data are depicted as mean +/− SEM. *N.S.* not significant, * p < 0.05, ** p < 0.01.

**Figure 3:**
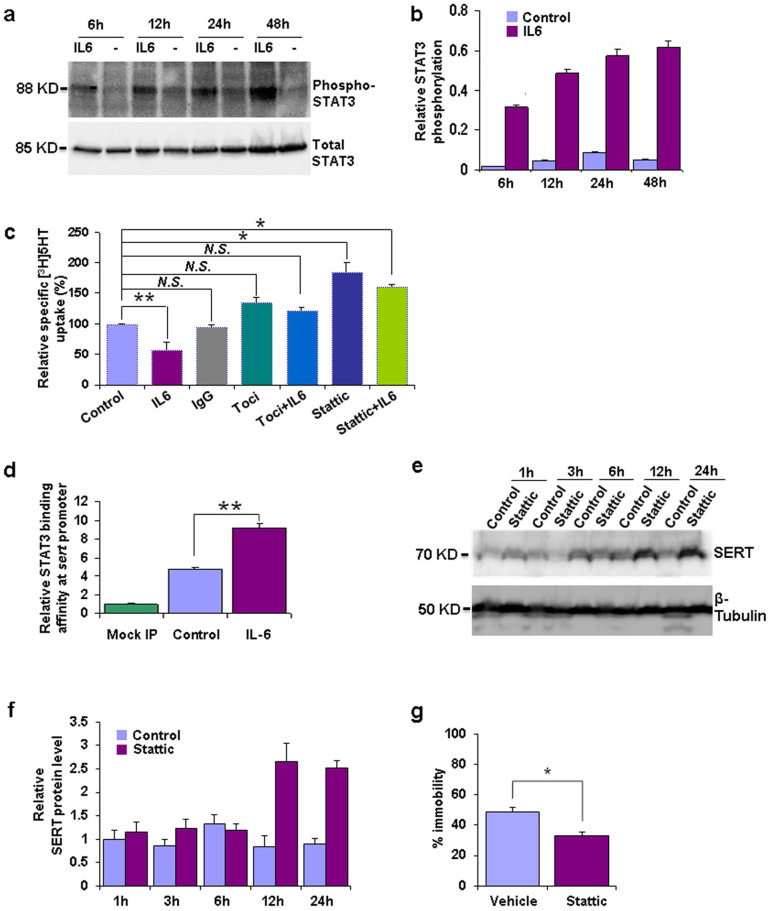
STAT3 controls SERT expression and function and modulates depression-like behavior. Time course of phospho-STAT3 and total STAT3 protein levels (Western Blot) in untreated control (**−**) and IL6 treated (IL6) JAR cells: (a) Western Blot image representative of three independent experiments with blot images cropped for comparison and (b) result of quantification (main effect of time *F*_(2,23)_ = 226.98, p = 0.0001, main effect of treatment *F*_(2,23)_ = 1796.69, p = 0.0001, time x treatment interaction *F*_(2,23)_ = 62.55, p = 0.0001; n = 3 per group). (c) Specific cellular [^3^H]5-HT uptake of JAR cells after 48 h of incubation with IL6, IgG1, tocilizumab (Toci), Stattic or combinations thereof and in untreated controls respectively. Relative specific [^3^H]5HT uptake values were quantified by the ratio of individual specific uptake values against that of control (p = 0.008, *F*_(6,22)_ = 7.17; n = 3–4 per group). (d) Chromatin immunoprecipitation (ChIP) analysis of STAT3 binding to the SERT promoter in untreated (control) and IL6 treated JAR cells (p = 0.0001, t*_(9)_* = 6.767, n = 6 per group). Time course of SERT hippocampal protein levels (Western Blot) of vehicle control and Stattic treated mice: (e) Western Blot image representative of three independent experiments with blot images cropped for comparison and (f) result of quantification (main effect of time *F*_(1,29)_ = 242.41, p = 0.0001, main effect of treatment *F*_(4,29)_ = 1496.39, p = 0.0001, time x treatment interaction *F*_(4,29)_ = 416.50, p = 0.0001; n = 3 per group). (g) Percentage of time spent immobile in the Forced Swim Test in vehicle control and Stattic treated mice 24 hrs after i.p. injection (p = 0.0028, t*_(7)_* = 4.487, n = 4 to 5 per group). Data are depicted as mean +/− SEM. *N.S.* not significant, * p < 0.05, ** p < 0.01; results of post-hoc pairwise comparisons are indicated in (c).

For statistical analyses of differences between two groups, data were tested for normality using the Kolmogorov–Smirnov test, followed by unpaired two-tailed Student's *t* tests (results depicted in Figures 1a–1f, 2a–2d, 2f–2g and 3g). For experiments involving more than two groups and/or more than one factor, one-way (results depicted in Figure 3c) or two-way ANOVA analysis (results depicted in Figures 2e, 3b and 3f) was carried out as appropriate. Post-hoc pairwise comparisons, with Bonferroni correction for multiple comparisons, were conducted where indicated. An α-level of 0.05 was adopted in all instances. All analyses were carried out using BioStat 2009 professional software (AnalystSoft Inc., Alexandria, VA, USA).

## Figures and Tables

**Figure 4:**
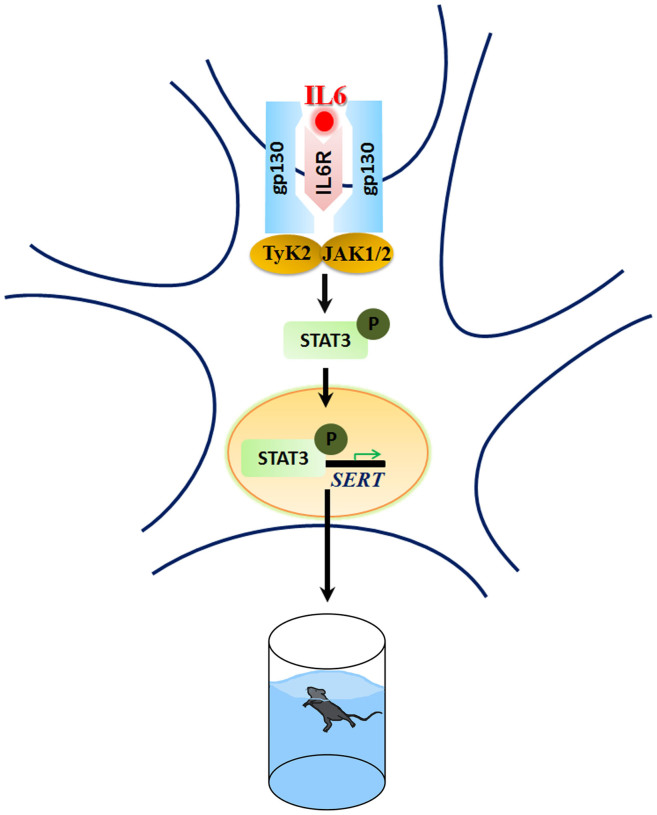
IL6-induced STAT3 signaling exerts a regulatory constraint on SERT expression, function and depression-like behavior in the mouse. Depicted is a schematic model explaining how STAT3 could mediate IL6-dependent regulation of serotonin transporter expression and depression-like behavior in mice. In this model, IL6 initiates its signaling through binding to IL6 receptor (IL6R), which activates downstream protein kinases including tyrosine kinase 2 (TyK2) and janus kinase 1/2 (JAK1/2), leading to the activation of STAT3 signaling through phosphorylation. Phosphorylated STAT3 translocates from the cytosol into the nucleus and binds to the conserved motif TTN5AA on the promoter region of the mouse SERT gene hereby regulating SERT transcription. Altered SERT expression level may contribute to the modulation of depression-like behavior in mice.
